# A bias in saccadic suppression of shape change

**DOI:** 10.1016/j.visres.2021.05.005

**Published:** 2021-09

**Authors:** Carolin Hübner, Alexander C. Schütz

**Affiliations:** a Allgemeine und Biologische Psychologie, Philipps-Universität Marburg, Marburg, Germany; b Center for Mind, Brain and Behaviour, Philipps-Universität Marburg, Marburg, Germany

**Keywords:** Transsaccadic perception, Intrasaccadic changes, Visual stability, Shape/form perception, Sensorimotor contingencies, Transsaccadic expectation/prediction

## Abstract

Processing of visual information in the central (foveal) and peripheral visual field is vastly different. To achieve a homogeneous representation of the visual world across eye movements, the visual system needs to compensate for these differences. By introducing subtle changes between peripheral and foveal inputs across saccades, one can test this compensation. We morphed shapes between a triangle and a circle and presented two different change directions (circularity decrease or increase) at varying magnitudes across a saccade. In a change-discrimination task, observers disproportionally often reported percepts of circularity increase. To test the relationship with visual-field differences, we measured perception when shapes were exclusively presented either in the periphery (before a saccade), or in the fovea (after a saccade). We found that overall shapes were perceived as more circular before than after a saccade and the more pronounced this difference was for a participant, the smaller was their circularity-increase bias in the change-discrimination task. We propose that visual-field differences have a direct and an indirect influence on transsaccadic perception of shape change. The direct influence is based on the distinct appearance of shape in the central and peripheral visual field in a trial, causing an increase of the perceptual magnitude of circularity-decrease changes. The indirect influence is based on long-term build-up of transsaccadic expectations; if a change is opposite (circularity increase) to the expectation (circularity decrease), it should elicit a strong error signal facilitating change detection. We discuss the concept of transsaccadic expectations and theoretical implications for transsaccadic perception of other feature changes.

## Highlights

• Intrasaccadic shape changes are detected more easily when circularity increases.
• Shape is perceived more circular presaccadically than postsaccadically.
• Transsaccadic expectations may determine intrasaccadic change detection.

## Graphical abstract

**Figure:**
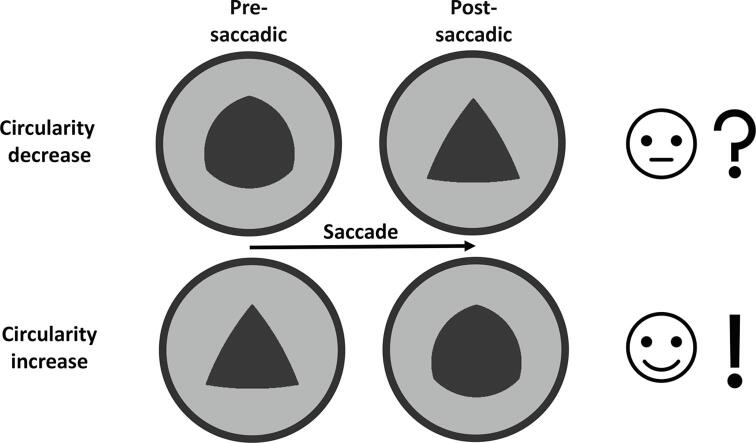

## 1 Introduction

The human visual system achieves a high visual resolution and a large field of view despite limitations in processing. The centre of the visual field, namely the fovea, provides highly detailed and relatively undistorted information due to the high density of cone photoreceptors (Oesterberg, 1935, Curcio et al., 1990) and an overrepresentation in the visual cortex (e.g., Dow et al., 1981, Azzopardi and Cowey, 1993, Dumoulin and Wandell, 2008). The periphery provides a large field of view, albeit with less detailed and more spatially distorted information (for reviews, see Strasburger et al., 2011, Rosenholtz, 2016). One function of saccades is to bring relevant objects into the fovea, which inevitably leads to a drastic change in the incoming low-level information due to this physiological disparity between foveal and peripheral processing. Given that human perception appears to be homogeneous and stable across eye movements, there must exist a mechanism eliminating such self-induced differences between pre- and postsaccadic information and previous research revealed a number of behavioural observations that might be the result of such a compensation mechanism.

One line of research reports relatively poor performance when externally induced visual changes at the moment of a saccadic eye movement have to be detected. This phenomenon is generally referred to as saccadic suppression and applies to a number of visual object properties such as spatial position (saccadic suppression of displacement, e.g., Bridgeman et al., 1975), object contour (Henderson, 1997, Demeyer et al., 2010), orientation (Henderson and Hollingworth, 1999, De Graef and Verfaillie, 2002, Grzeczkowski et al., 2020), object type and token (Henderson & Hollingworth, 2003), luminance (Henderson et al., 2008), and spatial frequency (Weiß et al., 2015). This elevation of change-detection thresholds during a saccade compared to fixation has been interpreted in the sense that the visual system has a tendency to discard small intrasaccadic changes and instead to maintain the assumption of a stable external world (e.g., MacKay, 1972). A prior assumption of external stability might hence be one measure by the visual system to compensate for self-induced discrepancies such as due to visual-field differences. Interestingly, saccadic suppression of change detection is not inevitable as accompanying signals can facilitate intrasaccadic change detection such as target blanking (e.g., Deubel et al., 1996, Deubel et al., 2002), changes in image size (McConkie & Currie, 1996), form changes (Demeyer et al., 2010), orthogonal target displacements (Wexler & Collins, 2014), and luminance or surface feature changes (Tas et al., 2012). Such visual events may break the stability assumption; but this and alternative explanations for their facilitative effect are still debated (e.g. Tas et al., 2012, Ziesche et al., 2017, Born, 2019) because a comprehensive charactarisation of the circumstances that lead to the facilitation is still missing.

Another line of research suggests that differences across the visual field are accounted for by the means of transsaccadic learning and transsaccadic predictions. Specifically, it has been shown that presaccadic stimuli appear more alike to a consistently accompanying postsaccadic stimulus after a relatively brief learning phase (e.g., Cox et al., 2005, Herwig and Schneider, 2014, Valsecchi and Gegenfurtner, 2016; for a review see Stewart, Valsecchi, & Schütz, 2020). Consistent with predictive coding theory (e.g., Rao and Ballard, 1999, Friston, 2009; for a review see de Lange et al., 2018), it has been suggested that a visual signal (Edwards et al., 2017), based on the recent transsaccadic experience, is generated upon processing of the presaccadic input and integrated with it leading to the biased appearance of the presaccadic stimulus. In essence, this line of research also suggests an experience-based mechanism (as any prediction should be based on experience) and this more specified predictive-coding mechanism might be likely candidates for how the visual system compensates for self-induced discrepancies and might as well be at the basis of intrasaccadic change detection (Ehinger et al., 2015).

To further characterise transsaccadic perception of change as well as to understand its relationship with appearance differences across the visual field, we investigated transsaccadic change perception a) of a key feature to mediate object constancy referred to as shape, form, or contour curvature (Kayaert et al., 2003, El-Shamayleh and Pasupathy, 2016), and b) with or without an accompanying signal that is known to facilitate change detection: a postsaccadic blank (Deubel et al., 1996). Additionally, we tested shape appearance pre- and postsaccadically, i.e., in the peripheral and central visual field. It is known from previous literature that the shape of geometric objects is perceived differently in the fovea and periphery (Baldwin et al., 2016, Coates et al., 2017, Valsecchi et al., 2018). Differences in appearance could have a direct effect on change perception as they could either perceptually increase or decrease the magnitude of a given physical discrepancy between pre- and postsaccadic inputs. For example, if shape is generally perceived as more circular in the periphery than in the fovea, intrasaccadic changes that increase circularity across a saccade should be reduced in perceived magnitude. Another and more indirect influence may come from transsaccadic predictions that are based on experienced transsaccadic contingencies. For example, in a scenario in which a less circular shape is predicted to follow after a saccade, a prediction error should be larger for more circular postsaccadic shapes and changes may be detected more easily.

## 2 Methods

The goal of this study was to investigate perception of shape changes across saccades and its interaction with perceptual differences between the peripheral and the foveal visual field. A second experiment was conducted to narrow down possible explanations for the direction of the observed bias in Experiment 1. Both experiments were divided into two parts: part A investigated transsaccadic shape change perception, and part B pre- (peripheral) and postsaccadic (foveal) shape appearance.

### 2.1 Participants

In Experiment 1, we tested 18 participants who were unaware of the purpose of the study of which one had to be excluded for not having executed a saccade in 98% of trials in part B. The data of 17 participants (10 females, 7 males; mean age = 23 years, range = 21–25 years) was used for analysis. In Experiment 2, a different group of 18 participants, who were unaware of the purpose of the study, was tested. Five of these participants had to be excluded. One did not complete both experimental parts. The four other excluded participants showed detection thresholds (in part A) that were unreasonably high (outside of our measurement range). That means that those participants did not achieve 75%-correct responses in at least one condition even with the largest shape changes we could apply. Thirteen participants (9 females, 4 males; mean age = 24 years, range = 20–28 years) remained for analysis. All participants were students of Marburg University, had normal or corrected-to-normal vision, and gave informed consent prior participation. The study was conducted in accordance with the principles of the Declaration of Helsinki (1964) and authorized by the local ethics committee of the psychology department at Marburg University (proposal number 2015–35 k).

### 2.2 Stimuli

**Fig. 1:**
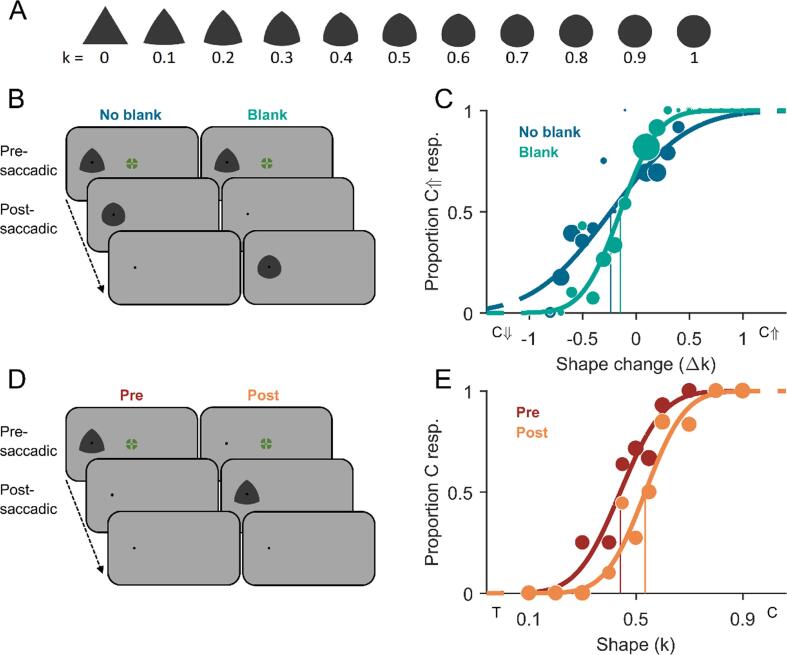
**Stimuli and methods of Experiment 1. A)** All shape stimuli with curvature index k going from 0 (triangular, T) to 1 (circular, C). Circumradii were adjusted to keep the covered area approximately constant across shapes. **B)** Schematic trial procedure of Experiment 1A showing a shape change of circularity increase across a saccade, either with a blank screen after the postsaccadic stimulus (no-blank condition) or before (blank condition). **C)** Example psychometric functions of one representative participant fitted to proportion circularity-increase (C↑) responses over shape changes tested (Δk) with negative deltas indicating circularity decrease (C↓) and positive deltas indicating circularity increase (C↑). Dark-blue data points (size scales with number of valid measurements) and fit represent the no-blank condition, and green represents the blank condition. Vertical lines indicate the points of subjective stability. **D)** Schematic trial procedure of Experiment 1B, in which participants had to compare the observed shape to the overall mean shape. Shape stimuli were either exclusively presented before the saccade in the peripheral visual field (presaccadic condition) or exclusively after the saccade in the central visual field (postsaccadic condition). **E)** Example psychometric functions of one representative participant fitted to proportion more-circular (C) responses over shapes tested (k) for the pre- (dark red) and postsaccadic condition (orange). Vertical lines indicate the points of subjective equality. A shape with k = 0.5 represents the true mean shape over all shapes. (For interpretation of the references to colour in this figure legend, the reader is referred to the web version of this article.)

**Fig. 2:**
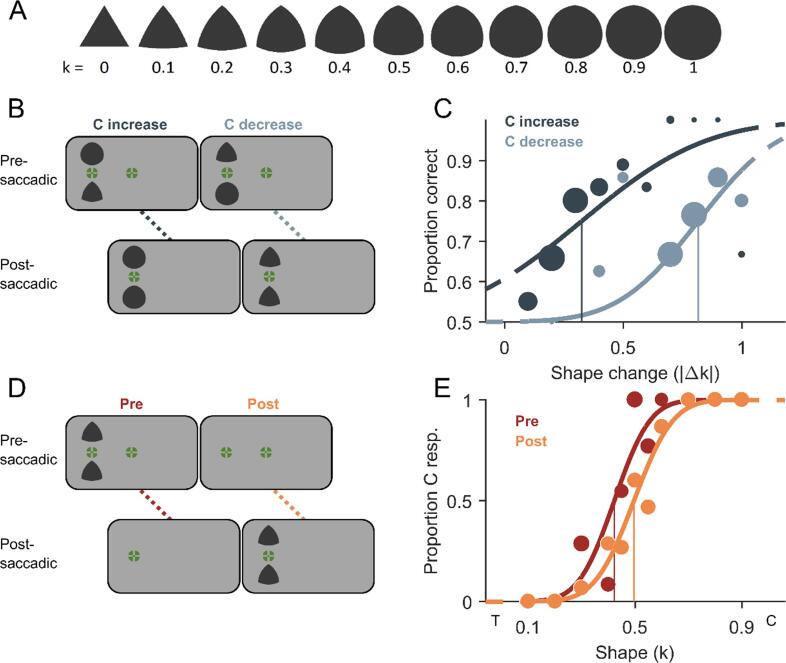
**Stimuli and methods of Experiment 2. A)** All shape stimuli with curvature index k going from 0 (triangular, T) to 1 (circular, C). Circumradii were kept constant across all shapes. **B)** Schematic trial procedure of Experiment 2A showing a shape change of circularity increase across a saccade in the left column and a change of circularity decrease in the right column. Two shapes were presented simultaneously and only one changed its shape resulting in two identical shapes after the saccade. The position of the shape change had to be indicated. **C)** Example psychometric functions of one representative participant fitted to proportion correct responses over absolute shape change magnitudes (|Δk|) for the change direction of circularity increase (dark grey) and circularity decrease (light grey). Data point size scales with the number of valid measurements and the vertical lines indicate detection thresholds (75% correct). **D)** Schematic trial procedure of Experiment 2B, in which participants had to discriminate the observed shape from the overall mean shape. The two identical shape stimuli were either exclusively presented before the saccade in the peripheral visual field (presaccadic condition) or exclusively after the saccade close to the central visual field (postsaccadic condition). **E)** Example psychometric functions of one representative participant for the pre- (dark orange) and postsaccadic condition (light orange). Conventions are identical to Fig. 1E. (For interpretation of the references to colour in this figure legend, the reader is referred to the web version of this article.)

The presaccadic fixation stimulus in Experiment 1 and the pre- and postsaccadic fixation stimuli in Experiment 2 were a combination of a bull’s-eye and crosshair (Thaler et al., 2013) with a diameter of 0.6° of visual angle, and of colour chosen randomly out of an array of colours generated in DKL colour-space (Derrington et al., 1984), with randomised polarity and isoluminance towards the grey background. The postsaccadic fixation stimulus in Experiment 1 was a black disk of 0.15° in diameter. Shape stimuli as depicted in Fig. 1A and Fig. 2A were similar to the ones used by Herwig et al., 2015, Paeye et al., 2018, and were generated based on an equilateral triangle which sides increased in curvature k in discrete steps of 0.1 going from k = 0 (full triangle) to k = 1 (full circle). Curvature k corresponds to the ratio of the circumradius and the radius of the three circles used for the geometrical construction of a Reuleaux triangle (Reuleaux, 1875). The circumradii of the shapes (k = 0, k = 0.1, …, k = 1) in Experiment 1 were 1.72°, 1.58°, 1.46°, 1.38°, 1.31°, 1.25°, 1.21°, 1.18°, 1.15°, 1.13°, 1.11° respectively. This was done to keep the area covered by each figure approximately the same for all shapes at 5885 pixels (Fig. 1A). In Experiment 2, all shape stimuli had a circumradius of 1.28° (Fig. 2A). All shape stimuli were dark grey (RGB: 56, 56, 56).

### 2.3 Design

Two experiments with two parts each were conducted in this study. In both experiments, intrasaccadic change detection was measured in part A and differences between pre- and postsaccadic appearance in part B. The crucial difference between Experiment 1 and 2 was that in Experiment 1, only one stimulus was shown before and after a saccade and that participants had to discriminate the direction of the intrasaccadic shape change (stimulus became more circular or more triangular) in part A. In Experiment 2, a pair of stimuli was shown before and after a saccade and participants had to discriminate which of the two stimuli changed its shape during the saccade in part A. In part B of both experiments, participants had to judge whether a shape perceived pre- or postsaccadically was either more circular or more triangular than the mean shape across all stimuli seen throughout the experiment (method of single stimuli; Morgan et al., 2000) independently of the number of shape stimuli presented in a trial. We used a staircase procedure in part A and the method of single stimuli in part B of both experiments. In Experiment 1A, two staircases were assigned to each change direction and blanking condition. One staircase started with the smallest possible change magnitude of 0.1 |Δk| and the other with the largest possible change magnitude of 1 |Δk|. The presaccadic shape was chosen randomly amongst all shapes that were not too close to the end of the shape range in respect to the applied change magnitude and direction. For example, if the change in a trial was assigned to −0.2 Δk, possible presaccadic shapes were all shapes except 0 and 0.1 k. If the change direction reported by the participant differed from the physical change direction the response was classified as a miss and the change magnitude was increased by a step size of 0.1 |Δk| for the next trial. If the reported change direction equalled the physical change direction, the response was classified as a hit and after two consecutive hits the change magnitude was decreased by the step size. Each staircase was running for 50 trials resulting in 400 trials in total for Experiment 1A. All conditions were tested interleaved and trial order was randomised. The design of Experiment 2A was similar to the one of Experiment 1A but the trial number for each staircase was 70 and there was no blanking condition, resulting in 280 trials for Experiment 2A. In Experiment 1B and 2B, 11 curvature values k (0.1, 0.2, 0.3, 0.4, 0.45, 0.5, 0.55, 0.6, 0.7, 0.8, 0.9) were tested for the presaccadic and postsaccadic condition with 15 repetitions each resulting in 330 trials. The two conditions were tested interleaved and trial order was randomised. In Experiments 1B and 2B participants completed a training of similar design as the main part of the experiment but without repetitions resulting in 22 trials. Training trials were excluded from analysis.

### 2.4 Equipment

For Experiment 1, stimuli were displayed on a VIEWPixx monitor at a 1920 × 1080 px resolution and a 120 Hz refresh rate. The display had a size of 51.5 × 29 cm and was viewed at a distance of 60 cm. The screen was calibrated to ensure a linear gamma correction and it had a luminance of 0.39, 54, and 105 cd/m^2^ for black, grey, and white pixels respectively. Eye movements were recorded with a desktop-mounted EyeLink 1000 (SR Research Ltd., Ontario, Canada) with a sampling rate of 1000 Hz. For Experiment 2, stimuli were presented using a back-projection setup, using a PROPixx projector (VPixx Technologies, Saint Bruno, QC, Canada), with a resolution of 1920 × 1080 px and a refresh rate of 120 Hz, projected onto a 91 × 51-cm screen from Stewart Filmscreen (Torrance, CA). Viewing distance was 106 cm. The screen was calibrated to ensure a linear gamma correction and to minimize the central hot spot, and it had a luminance of 2.07, 71, and 140 cd/m^2^ for black, grey, and white pixels respectively. Eye movements were recorded using a tower-mounted EyeLink 1000 Plus (SR Research Ltd., Ontario, Canada), with a sampling rate of 1000 Hz. Experimental software and analysis were written in MATLAB (Mathworks, Natick, MA, USA), using Psychophysics Toolbox (Brainard, 1997, Pelli, 1997) for stimulus display and the EyeLink Toolbox (Cornelissen et al., 2002) for eye tracker operation. Participants responded using a standard keyboard (vertical plus-sign button on number pad for towards-triangular or more-triangular responses and horizontal zero button on number pad for towards circular or more-circular responses; up- and down arrow keys for upper/lower responses in Experiment 2A) and their head position was stabilised using a forehead- and chinrest.

### 2.5 Procedure

Participants started each trial by pressing the space bar while fixating a central fixation stimulus. In Experiment 1A, the presaccadic shape appeared to the left or right at an eccentricity of 15° of visual angle on the horizontal axis after a duration jittered between 750 and 1500 ms. The participants were instructed to execute a saccade toward the centre of the peripheral shape, which was marked by a black dot (Fig. 1B). The fixation stimulus at screen centre remained on screen for additional 200 ms or until a saccade was detected. A trial was aborted when no saccade was detected within 1.8 s after saccade target onset. Upon saccade detection, the shape stimulus was replaced either immediately (no-blank condition), or removed (the black dot remained) for 200 ms (blank condition) and then replaced by the postsaccadic shape stimulus. The postsaccadic stimulus was displayed for half of the duration of the presaccadic stimulus in a given trial of the blank condition, and plus 30 ms in a given trial of the no-blank condition (to compensate for the time during the saccade). The extra time between trial start and response screen onset in a blank trial (due to the postsaccadic blank) was added to the no-blank condition but after the postsaccadic stimulus presentation; i.e., the central dot at saccade target position remained on screen for 170 ms. Finally, the blank screen prompted participants to give a response by button press, indicating whether the change was perceived as going toward a more triangular shape or toward a more circular shape. A high tone was played when the gaze behaviour in that trial was incorrect according to the criteria stated for trial exclusions below. A low tone was played when the response for the change direction was incorrect. No tone was played and the trial ended immediately after the response was given when gaze behaviour and response were correct.

In the trial procedure of Experiment 1B (Fig. 1D), either a shape stimulus plus central dot (presaccadic condition), or solely the black dot (postsaccadic condition) appeared presaccadically at an eccentricity of 15° of visual angle on the horizontal axis after a duration jittered between 750 and 1500 ms from trial start. Upon saccade detection, the presaccadic stimulus was either reduced to the uninformative central dot (presaccadic condition: shape information only presaccadically) or the shape stimulus was added (postsaccadic condition: shape information only postsaccadically). Postsaccadic-stimulus presentation duration equalled half the participant’s median presaccadic-stimulus presentation duration over all completed trials of the presaccadic condition plus 30 ms. After the postsaccadic shape stimulus offset, the black target dot remained on screen for another 170 ms. The consecutive blank screen prompted participants to give a response by button press indicating, whether the perceived shape was more triangular or more circular than the mean of all shapes seen thus far. There was no feedback given on the correctness of the response but a high tone was played for irregular gaze behaviour similarly to part A. In the 22 training trials, both kinds of feedback were given. The order of completion of part A and B was counterbalanced across participants and data was collapsed across order (AB or BA) as analysis revealed no effect of order.

The procedures of Experiment 2A (Fig. 2B) and B (Fig. 2D) were similar to the one of Experiment 1A and B respectively, except that two shape stimuli (without central black dots) were shown pre- and postsaccadically, one below and one above a second fixation stimulus centred between them, with a distance of 2.5° between the centre of one shape and the centre of the second fixation stimulus. Eccentricity from the first fixation stimulus was ± 5° on the horizontal axis. In Experiment 2A, the two shapes were always different presaccadically and identical postsaccadically and responses were given to indicate the location of the shape change (top or bottom). The presentation duration of the postsaccadic stimuli equalled half the presentation duration of the presaccadic stimuli on a given trial. In Experiment 2B, the presentation duration of the postsaccadic stimuli equalled half the participant’s median presentation duration of the presaccadic stimuli over all completed trials of the presaccadic condition and the empty response screen followed the postsaccadic shape stimuli offset immediately.

### 2.6 Eye-movement analysis and trial exclusions

For eye-movement data analysis saccades were detected offline using the EyeLink 1000 algorithm (velocity threshold = 22°/s, acceleration threshold = 3800°/s^2^). Saccade onset was defined as the first sample after saccade-target onset in which a saccade was detected; likewise, saccade offset was defined as the last sample after saccade onset in which a saccade was detected. Postsaccadic landing position was taken at the point of saccade offset. Saccade latency was defined as the time (resolution of 1 ms) between saccade-target onset and saccade onset. Results regarding saccade latencies can be found in the Supplementary material.

Trials, which contained blinks in the time between 300 ms to saccade-target onset and response-screen onset, trials, in which the switch between pre- and postsaccadic stimulus was not achieved in the time of the saccade (e.g., due to small, consecutive saccades instead of one large saccade), and trials, in which not the full sequence of events was run through were excluded from analysis. We further excluded trials with saccade latencies below 50 ms or above 600 ms. Further trials were excluded when gaze position deviated more than 2° on the horizontal axis or more than 1.5° on the vertical axis, from saccade target centre in the time between saccade landing and shape stimulus offset. In total, 11 ± 10% (mean ± standard deviation, over participants and conditions) of trials were excluded from Experiment 1A, 17 ± 10% from Experiment 1B, 5 ± 4% from Experiment 2A, and 10 ± 9% from Experiment 2B.

### 2.7 Psychophysical analysis

To obtain psychometric functions for each participant for Experiments 1A (Fig. 1C), perceptual choices were converted into proportion circularity-increase responses for each shape change tested. A cumulative Gaussian was fitted to the data using psignifit 4.0 toolbox (Schütt et al., 2016). The point of subjective stability (PSS) was estimated as the magnitude and direction of shape change (Δk) corresponding to 50% circularity-increase responses. A negative PSS indicates a bias for reporting circularity-increase shape changes. The just-noticeable difference (JND) was defined as the standard deviation of the cumulative Gaussian, with a lower JND indicating higher precision for shape-change discrimination.

Similarly to the data analysis for Experiment 1A, responses in Experiment 1B and 2B were converted into proportion more-circular (than the mean shape) responses for each shape tested, and psychometric functions were fitted (Fig. 1E, Fig. 2E). The point of subjective equality (PSE, parameter equivalent to PSS) was estimated as the degree of curvature (k) corresponding to 50% more-circular responses. A PSE above 0.5 indicates a bias for perceiving shapes as more triangular; accordingly, a PSE below 0.5 indicates a bias to perceive shapes as more circular. The just-noticeable difference (JND) was defined as the standard deviation of the cumulative Gaussian, with a lower JND indicating higher precision for shape discrimination.

Perceptual choices in Experiment 2A were converted into proportion correct responses for each shape change magnitude tested for both change-direction conditions. A cumulative Gaussian starting at chance level of 50%-correct responses was fitted to the data for each participant using psignifit 4.0 toolbox (Schütt et al., 2016). The detection threshold was estimated as the absolute magnitude of shape change (|Δk|) necessary for a participant to reach 75%-correct responses. A lower threshold indicates higher sensitivity to the corresponding shape-change direction (Fig. 2C). For all statistical tests, the alpha value was set to 0.05 and t-tests were two-tailed.

## 3 Results

### 3.1 Experiment 1 – Shape perception biases and blanking effect

**Fig. 3:**
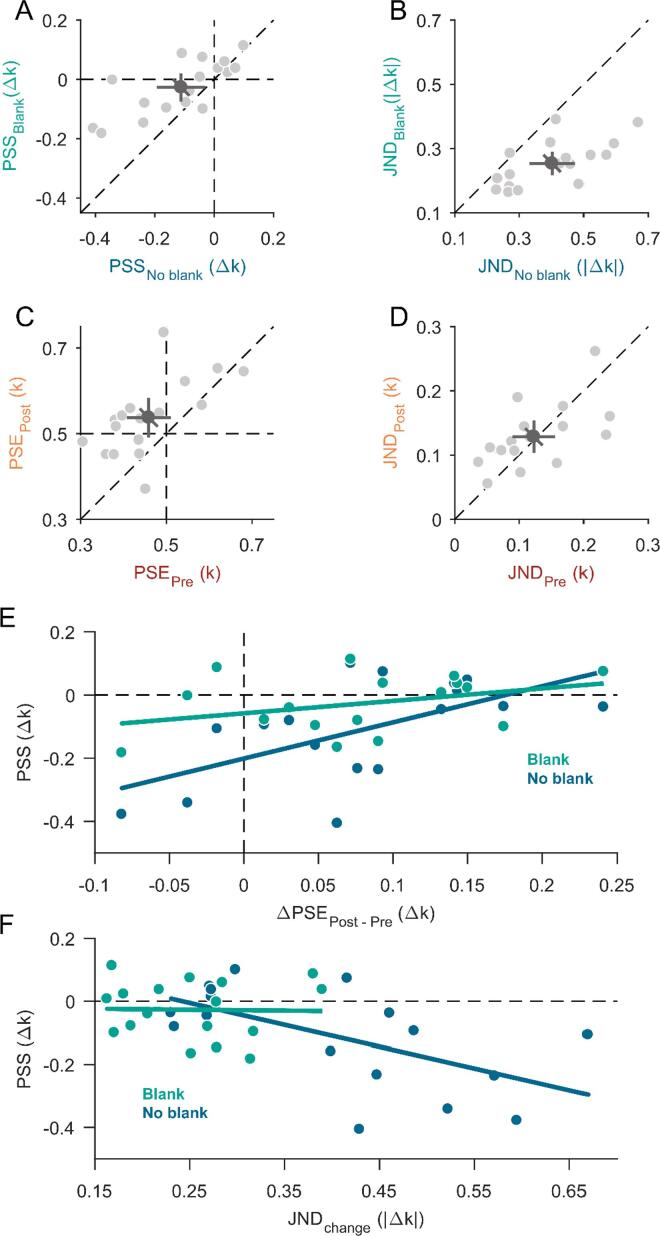
**Results from Experiment 1. A)** Scatter plot for all points of subjective stability (PSS) compared between the no-blank condition (horizontal axis) and blank condition (vertical axis) of Experiment 1A. Data points left from the dashed vertical line or below the dashed horizontal line (negative PSS) indicate a bias for circularity-increase changes. **B)** Scatter plot for just-noticeable differences (JNDs) compared between the no-blank condition (horizontal axis) and blank condition (vertical axis) of Experiment 1A. Data points below the diagonal dashed line indicate that participants were more precise in the blank condition. **C)** Points of subjective equality (PSE) compared between pre- and postsaccadic condition in Experiment 1B. Data points above the dashed diagonal line indicate a less circular appearance in the postsaccadic condition compared to the presaccadic condition. **D)** Just-noticeable differences (JNDs) compared between pre- and postsaccadic condition in Experiment 1B. **A-D)** Data points on the dashed diagonal line indicate no difference between conditions. Light-grey dots represent individual participant data and the dark-grey dot indicates the overall mean. The error bars indicate 95%-confidence intervals within each condition (cardinal bars) or between conditions (oblique bar). **E)** The effect of individual perceptual differences between pre- and postsaccadic vision (difference of PSEs from Experiment 1B, horizontal axis) on the bias in the change-discrimination task (PSS from Experiment 1A, vertical axis) separately for the blank (green) and no-blank condition (dark blue). The more positive a PSE difference, the stronger a bias for perceiving shapes as more circular presaccadically and the more negative a PSS, the stronger was the bias for circularity-increase changes. Linear regression fits for each blanking condition are represented by the coloured solid lines. **F)** The effect of individual precision (JNDs, horizontal axis) on the bias (PSS, vertical axis) in the change-discrimination task of Experiment 1A separately for the blank (green) and no-blank condition (dark blue). Increasing JNDs indicate decreasing precision and the more negative a PSS the more of a circularity-increase bias was observed. Linear regression fits for each blanking condition are represented by the coloured solid lines. (For interpretation of the references to colour in this figure legend, the reader is referred to the web version of this article.)

In Experiment 1A, we increased or decreased the circularity of the shape stimulus during the saccade and asked participants to report the perceived direction of the change. The mean point of subjective stability (PSS) was −0.11 ± 0.16 Δk for the no-blank condition and −0.03 ± 0.09 Δk for the blank condition (Fig. 3A). The mean PSS for the no-blank condition was significantly different from zero (t(16) = -2.89, p = 0.011) indicating that participants had a bias to report transsaccadic shape changes of increasing circularity. A tendency for such a bias was also observed in the blank condition but it was not significantly different from zero (t(16) = -1.21, p = 0.243) and the difference between PSS for the no-blank and blank condition was significant (t(16) = -3.20, p = 0.006). The mean just-noticeable difference (JND) for shape change discrimination in Experiment 1A was 0.40 ± 0.14 |Δk| for the no-blank condition and 0.25 ± 0.07 |Δk| for the blank condition (Fig. 3B). JNDs were significantly different (t(16) = 5.85, p < 0.0001) between blanking conditions. In sum, participants were significantly more precise (JNDs) and more accurate (PSS) at discriminating shape-change direction in the blank condition compared to the no-blank condition. This result indicates a blanking effect for shape changes.

In Experiment 1B, we measured the appearance of the shapes presaccadically in the periphery and postsaccadically in the fovea. The mean point of subjective equality (PSE) in Experiment 1B was 0.46 ± 0.10 k for the presaccadic condition and 0.54 ± 0.09 k for the postsaccadic condition (Fig. 3C). The mean PSE for the presaccadic condition (t(16) = -1.70, p = 0.109) and postsaccadic condition (t(16) = 1.72, p = 0.107) were both not significantly different from the true mean of the shape stimuli of 0.5 k, but significantly different from each other (t(16) = -3.93, p = 0.0012). This indicates that participants perceived the shapes on average as more circular presaccadically in the periphery and as more triangular postsaccadically in the fovea. The mean just-noticeable difference (JND) in Experiment 1B was 0.13 ± 0.05 k for the presaccadic condition and 0.12 ± 0.06 k for the postsaccadic condition (Fig. 3D). The difference in JNDs between the pre- and postsaccadic condition was not significant (t(16) = 0.43, p = 0.675), which indicates that participants were equally precise at discriminating shapes from the mean shape pre- and postsaccadically^1^.

Most interestingly, the overall bias in the change discrimination task (PSS in Experiment 1A) cannot be explained by a direct influence of appearance differences between presaccadic peripheral and postsaccadic foveal vision (differences between PSEs in Experiment 1B). In fact, the more circular appearance in pre- compared to postsaccadic vison should increase the perceived change magnitude for circularity-decrease but participants showed a bias to report circularity-increase changes instead (see also Figure S1). To obtain a more detailed insight into the relationship between appearance differences and change discrimination biases, we analysed the impact of individual differences between pre- and postsaccadic shape perception (differences between pre- and postsaccadic PSEs of Experiment 1B) on participants’ biases (PSS of Experiment 1A) in the change discrimination task (Fig. 3E). A positive correlation between the PSE differences and PSS was observed for the no-blank condition (slope m = 1.14, $p_{m}$ = 0.017, y-intercept n = -0.20, $p_{n}$ < 0.001, r^2^ = 0.33) and a similarly oriented but non-significant relationship for the blank condition (m = 0.44, $p_{m}$ = 0.172, n = -0.06, $p_{n}$ = 0.09, r^2^ = 0.12). The positive slope may suggest that perceptual differences between pre- and postsaccadic perception do have a direct influence on transsaccadic change perception. Participants who perceived the shapes on average as more triangular postsaccadically than presaccadically (positive PSE differences in Fig. 3E) showed a smaller bias to disproportionally often report changes with circularity increase (PSS values closer to zero in Fig. 3E). Above and beyond this direct influence, the significantly negative intercept for the no-blank condition again shows that there was a bias to report circularity increase more often. The origin of this bias remains an open question that we will address in the discussion.

As we observed that the overall circularity-increase bias was reduced in the blank condition, where participants also were more precise (lower JNDs in part A) we further tested whether the magnitude of the bias was related to the precision across participants. The negative correlation for the no-blank condition (m = -0.70, $p_{m}$ = 0.015, n = 0.17, $p_{n}$ = 0.131, r^2^ = 0.33) shown in Fig. 3F indicates that lower precision in change discrimination was accompanied by a larger bias. The smaller variance across JNDs in the blank condition did not seem to affect the bias (m = -0.01, $p_{m}$ = 0.981, n = -0.02, $p_{n}$ = 0.811, r^2^ < 0.01). These results indicate that participants who had a harder time discriminating intrasaccadic shape changes (showed greater JNDs) benefited most from the circularity-increase change direction in terms of detectability (more negative PSS). Similarly, when there was a postsaccadic blank (i.e., JNDs were low) both change directions were equally well detectable.

### 3.2 Experiment 2 – Perceptual bias for circularity-increase changes

**Fig. 4:**
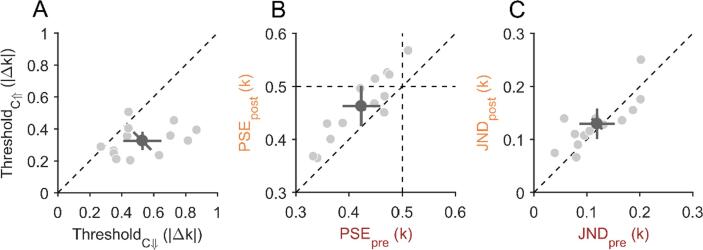
**Results from Experiment 2. A)** Scatter plots for all detection thresholds compared between the circularity-decrease (C↓, horizontal axis) and circularity-increase condition (C↑, vertical axis) of Experiment 2A. Data points below the diagonal dashed line indicate lower thresholds for the circularity-increase change direction. **B)** Points of subjective equality (PSE) compared between pre- and postsaccadic condition in Experiment 2B. PSEs below 0.5 indicate a participant’s bias for disproportionally often judging shapes to be more circular. Data points above the dashed diagonal line indicate a less circular appearance in the postsaccadic condition compared to the presaccadic condition. **C)** Just-noticeable differences (JNDs) compared between pre- and postsaccadic conditions in Experiment 1B. Data on the diagonal dashed line indicate that participants were equally precise in both conditions. **A-C)** Light-grey dots represent individual participant data and the dark-grey dot indicates the overall mean. The error bars indicate 95%-confidence intervals within each condition (cardinal bars) or between conditions (oblique bar).

In Experiment 1A, we observed a bias for circularity-increase reports that led to a shift of the PSS. Theoretically, this bias alone could be interpreted as a perceptual bias, a response bias for one response alternative or even a response bias for one of the two response keys. However, the correlation between the bias in Experiment 1A and the differences in pre- and postsaccadic appearance in Experiment 1B (Fig. 3E) cannot be explained by any response bias and strongly suggest a perceptual bias. To provide further evidence that this was a perceptual bias and not a mere response bias for one response alternative or for one response key, we performed Experiment 2. Here, a pair of shape stimuli was shown before and after the saccade and only one stimulus changed its shape during the saccade. Participants had to report which of the two stimuli was changed. The mean detection threshold was 0.53 ± 0.20 |Δk| for the circularity-decrease condition and 0.33 ± 0.10 |Δk| for the circularity-increase condition (Fig. 4A). Detection thresholds were significantly lower when shapes increased in circularity across a saccade compared to a circularity decrease (t(12) = 3.97, p = 0.002). This result replicates the change-direction bias observed in Experiment 1A and rules out the possibility of a response bias, meaning that participants not only reported but also perceived circularity-increase changes disproportionally often. In other words, the most likely explanation for the circularity-increase bias in PSSs in Experiment 1 are the lower detection thresholds for circularity increases compared to circularity decreases in Experiment 2.

Similarly to Experiment 1B, we measured the appearance of the shapes presaccadically in the periphery and postsaccadically near the fovea in Experiment 2B. The mean point of subjective equality (PSE) from Experiment 2B was 0.42 ± 0.06 k for the presaccadic condition and 0.46 ± 0.06 k for the postsaccadic condition (Fig. 4B). The mean PSE of the presaccadic condition was significantly different from the true mean of 0.5 k (t(12) = -4.68, p < 0.001), but that of the postsaccadic condition was not (t(12) = -2.14, p = 0.053). Mean PSEs of both conditions were significantly different from each other (t(12) = -5.57, p < 0.001). This replicates the finding from Experiment 1B that participants perceived the shapes on average as more circular presaccadically in the peripheral visual field and as more triangular postsaccadically near the central visual field. The mean just-noticeable difference (JND) from Experiment 2B was 0.12 ± 0.05 k for the presaccadic condition and 0.13 ± 0.05 k for the postsaccadic condition (Fig. 4C). The difference in JNDs between the pre- and postsaccadic condition was not significant (t(12) = -1.11, p = 0.291), which indicates that participants were equally precise at discriminating shapes from the mean shape pre- and postsaccadically, as it was the case in Experiment 1B.

## 4 Discussion

In this study, we investigated the perception of shape changes during saccadic eye movements and its relationship to pre- and postsaccadic appearance of shape. Our results confirm that transsaccadic perception of shape changes underlies the same effects that apply to similar (Deubel et al., 2002, Experiment 3; Grzeczkowski, van Leeuwen, et al., 2020) and other object features: performance at intrasaccadic change detection was relatively poor under normal conditions (no-blank condition) and an accompanying postsaccadic blank facilitated change detection (Fig. 3B). On the other hand, shape changes seem to be extraordinary (but see section 4.3 Transsaccadic expectations and other feature changes) as the direction of change influenced change detectability under normal conditions such that changes with increased circularity were detected more often than changes with decreased circularity (Fig. 3A & Fig. 4A). We could rule out that this was due to a simple response bias for choice category as we implemented a criterion-free paradigm in Experiment 2. We can also rule out the possibility that the bias in shape-change discrimination might be due to changes in size (circumradius) or covered area between shapes as we fixed one of these metrics in each experiment (Fig. 1A & Fig. 2A).

We found that shape appearance was distinct between pre- and postsaccadic perception such that shapes generally appeared more circular presaccadically in the peripheral visual field (at 15° in Experiment 1, and at 5° in Experiment 2) compared to postsaccadically in the fovea (Experiment 1, Fig. 3C) or near it (Experiment 2, Fig. 4B). This means that the differences in appearance cannot directly explain the overall bias in the perception of shape-changes in terms of a perceptual increase of the circularity-increase change magnitude. In fact, a more circular appearance of shape in the periphery should reduce the magnitude of a shape change that increased circularity across a saccade. Our finding on appearance differences may be compared to other findings on appearance differences between peripheral and foveal vision. For example, it was shown that stimulus size appears smaller in the periphery (Newsome, 1972), and numerosity (number of dots in a dot cloud) appears lower in the periphery (Valsecchi et al., 2013; but see Hübner & Schütz, 2017). What determines less triangular appearance in the periphery might be related to what causes the size or numerosity reduction (see also section 4.2 Shape across the visual field). However, we want to emphasise that our and these other findings on visual-field differences in the appearance of visual features are not directly comparable. Pre- and postsaccadic perception are not equivalent to mere perception at the periphery and fovea. This may especially be the case for spatial features such as spatial frequency, numerosity, or shape since it has been shown that the preparation of a saccade abolishes visual crowding (Harrison et al., 2013), and enhances spatial resolution (Li et al., 2016, Li et al., 2019). Measuring pre- and postsaccadic appearance represents a more complete account in regard to transsaccadic perception. This may be especially evident considering that presaccadic appearance likely results from an integration of presaccadic sensory information with the prediction for the postsaccadic outcome (e.g., Herwig et al., 2015, Valsecchi and Gegenfurtner, 2016). This integration will inevitably make pre- and postsaccadic appearance more similar.

We further found that inter-individual variations of pre- and postsaccadic differences (differences between PSEs of Experiment 1B) systematically influenced shape-change perception (shifts in the PSS of Experiment 1A) as shown by a significant positive correlation between the two (Fig. 3E). This correlation can only be based on a perceptual bias and cannot be explained by any response bias. Taken together, our results may suggest that visual-field differences have a direct and an indirect influence on transsaccadic perception of shape changes. The direct influence is based on the distinct appearance of shape pre- and postsaccadically; if a shape appears more circular before than after the saccade, shape changes with circularity increase should have a smaller perceptual magnitude and be missed more easily than changes with circularity decrease. However, the perceived magnitude of a shape change only seems to play a subsidiary role as we found an overall bias in the opposite direction. Change direction predominantly affected shape-change perception and this may be due to visual field differences as well, but indirectly. We suggest that a life-time experience of appearance changes leads to the build-up of transsaccadic expectations^2^ that serve as a measure for the visual system to evaluate perceptual evidence for or against external stability. One might infer from the pre- and postsaccadic appearance differences of shape that the typical experience of the visual system should be a circularity decrease in saccade direction (perceived circularity is higher presaccadically than postsaccadically) and that similar experiences with real-world shapes have formed the expectation responsible for the observed bias. The principal assumption we make is that a contradiction of such an expectation, i.e., a circularity-increase change should be evaluated as strong evidence against stability and facilitating change detection, leading to the overall bias for circularity increase. It seems likely that participants, who relied more strongly on expectations than others benefited more from a circularity-increase change i.e., showed a stronger circularity-increase bias and also showed smaller differences in pre- and postsaccadic appearance (as presaccadic appearance would more strongly be influenced by the prediction).

According to formulations in predictive coding theory (Feldman and Friston, 2010, Bastos et al., 2012), participants who rely more on predictions and down-weight predictions errors should also show lower sensory precision. Evidence following this line comes from the correlation of individual differences in change-discrimination precision (JNDs in Experiment 1A) with individual bias strength (Fig. 3F). Participants who were less precise might have down-weighted predictions errors (in classical terms: they had a stronger assumption of stability) and hence, tolerated larger discrepancies between pre- and postsaccadic information. Those participants revealed a larger circularity-increase bias, which suggests that this change direction caused prediction errors strong enough to make the external change detectable despite the down-weighting. It should, however, be mentioned that increased change-discrimination precision might also be due to larger pre- and postsaccadic appearance differences in those participants, which, potentially, facilitated the detection of circularity-decrease changes more than it impaired the detection of circularity-increase changes (Figure S2). Given trials with a postsaccadic blank, JNDs were overall smaller, a circularity-increase bias was nullified, and there was no more correlation between individual precision and bias strength. This pattern of results would be expected if a postsaccadic blank already caused a maximally large prediction error (in classical terms: abolished the stability assumption) and there would have been nothing left for strong evidence coming from a circularity-increase change to add.

### 4.1 Transsaccadic expectations

A striking commonality amongst all visual events that improve intrasaccadic change detection performance is that they are unexpected with respect to what can be learned from every-day transsaccadic experience (O’Regan & Noë, 2001). For example, discrepancies between saccade landing position and postsaccadic target position (referred to as retinal error) in parallel to saccade direction are “experienced” by the visual system to a greater degree due to an individual’s natural landing variability (van Opstal and van Gisbergen, 1989, Niemeier et al., 2003). On the contrary, orthogonal displacements place saccade targets outside the typically experienced, oval window of saccade landing variability (Niemeier et al., 2007, Wexler and Collins, 2014, Atsma et al., 2016). Such an orthogonal error should contradict what could be learned from every-day experiences and therefore facilitate detection of a change. A second example may be that visual disruption that can be anticipated by the visual system, such as the visual blank caused by blinks, does not facilitate transsaccadic change detection in contrast to externally imposed blank periods (Deubel et al., 2004). In general, it seems that less frequently experienced discrepancies reach consciousness and facilitate change detection while more frequently experienced discrepancies fail to reach consciousness and change detection is suppressed. Similarly, we show that, due to pre- and postsaccadic appearance differences, the typical transsaccadic experience of shape is that circularity decreases in saccade direction. Appearance differences experienced throughout life might form transsaccadic expectations about the typical magnitude and, importantly, the typical direction of change. Hence, changes that are opposite to the expected change direction should lead to an increased error or may be taken as strong evidence for a change in the external world, reducing the impact of an assumption of external stability^3^.

Change detection facilitation due to a specific change direction has, until now, only been reported for saccade target displacements (McConkie and Currie, 1996, Niemeier et al., 2007, Wexler and Collins, 2014, Atsma et al., 2016, Souto et al., 2016). The underlying concepts of two models (Niemeier et al., 2003, Atsma et al., 2016) that can explain such a facilitation for target displacements orthogonal- compared to parallel to saccade direction may be similar to what was first suggested by MacKay (1972); namely, a dichotomy between the two possible scenarios of either an external change or no external change for or against which evidence can be evaluated based on transsaccadic expectations. Transsaccadic predictions appear to be the measure for the visual system by that transsaccadic expectations (experience-based knowledge on transsaccadic contingencies) become effective. To give a simplified example, if the visual system has learned that shapes typically become more triangular across a saccade, the visual signal that gets generated for, e.g., a medium shape of k = 0.5 in the periphery, should be of a more triangular shape (e.g., k = 0.1) and fed back to lower visual areas before the postsaccadic information arrives. The discrepancy (also referred to as prediction error in predictive coding) between this prediction (that relies on presaccadic sensory information and transsaccadic expectations) and the actual postsaccadic shape should be larger when the postsaccadic shape is more circular (e.g., k = 0.7), than when the postsaccadic shape information would be more triangular (e.g., k = 0.2), and a larger error should facilitate change perception. The overall bias we found for circularity increase suggests that a transsaccadic prediction (more triangular), rather than the presaccadic information (more circular), is compared to the postsaccadic information. An integration of the prediction with the presaccadic input may take place subsequently and, possibly, only when no postsaccadic input was available e.g., when presaccadic appearance is tested. Models on intrasaccadic change perception (e.g. Atsma et al., 2016) should incorporate transsaccadic predictions that are specific to the learned transsaccadic contingencies of the feature at hand.

Alternative theoretical accounts for intrasaccadic change detection have been proposed to explain benefits from target blanking and are based on the potential benefit provided by the extra amount of input-free time during the blank period, enabling either a sufficient read-out of the presaccadic target information, or providing sufficient time to process upcoming postsaccadic information outside the time window of suppression of contrast sensitivity (e.g. Zimmermann et al., 2013, Ziesche et al., 2017). Such accounts fail to offer a potential explanation for our shape-change direction bias, and a row of other findings on transsaccadic change perception. For example, the improvement of displacement detection due to accompanying object-form changes (Demeyer et al., 2010) or other accompanying feature changes (Tas et al., 2012), or a stronger blanking effect for children compared to adults (Stewart, Hübner, & Schütz, 2020). Overall, an account based on evidence evaluation for or against a stable transsaccadic percept appears to be the more comprehensive theory for visual stability across saccades and, with consideration of feature-specific transsaccadic expectations, the most likely theory behind our findings.

### 4.2 Shape across the visual field

Assuming that transsaccadic expectations led to the observed circularity-increase bias, it should be evaluated what the particular character of the typically experienced saccade-induced contingency is, that could have led to such an expectation. To do that, we need to evaluate what determines shape information in the periphery compared to the fovea. We know that the peak of the spatial contrast sensitivity function is shifted to lower spatial frequencies in the periphery compared to the fovea (e.g., Rovamo et al., 1992), which may imply that two intersecting lines or edges become less visible in the periphery the smaller the angle separating them (the sharper a corner). In addition, spatial localisation of available visual information is more difficult in the periphery (Rentschler and Treutwein, 1985, Levi and Klein, 1986, Hess and Hayes, 1994), potentially leading to distorted shape information and edges that are spatially misaligned. Illustrations of the approximated distortion in low-level peripheral processing for shape can be found in the work by Valsecchi et al. (2018), who manipulated overlapping geometric shape stimuli using an image-manipulation algorithm that was designed to simulate all aspects of low-level peripheral processing (Eidolon factory, Koenderink et al., 2017). Taken together, these studies point at two key properties that might determine low-level shape information across the visual field: spatial detail (sharpness) and shape continuity (degree of distortion).

Our finding that shapes are perceived as more circular in the periphery (Fig. 3C & Fig. 4B) could be caused by the limited processing capacity of both of these properties. Fine corners were either not represented for the lack of visual detail or they were mis-localised to some degree that gave the impression of not being part of the figure/shape. Alternatively, they might be removed in order to rectify spatial disarray in the periphery. For instance, perceptual illusions such as the honeycomb illusion (Bertamini et al., 2019, Bertamini et al., 2016) may indicate that fine visual detail is reasonably well resolvable and localisable in the periphery but becomes less visible for the sake of a simple geometrical shape representation. Consistently with this interpretation, Valsecchi and colleagues (2018) showed that irregular shapes appear less irregular in the periphery than in the fovea; and it is known that feedback information is at the basis of shape perception (e.g., Hupé et al., 1998, Murray et al., 2002, Kok and de Lange, 2014). This would mean that for our intermediate shapes, even when corners could be resolved and located presaccadically they might have been rationalised to represent a circle as a less ambiguous shape.

In conclusion, all possibilities predict that spatial detail such as corners should rather add to an object’s shape across a saccade than disappear. This may be due to the lower resolution, higher localisation uncertainty, or some mid-level rationalisation for circles in the periphery. Given that this low- or mid-level discrepancy was measurable between pre- and postsaccadic appearance (Fig. 3C & Fig. 4B) we cannot identify whether transsaccadic expectations were learned from appearance differences or from lower-level differences.

### 4.3 Transsaccadic expectations and other feature changes

Shape is known to mediate object constancy (Kayaert et al., 2003, El-Shamayleh and Pasupathy, 2016) and may even be one of the most relevant properties for the deduction of laws following from sensorimotor contingencies (e.g., Koenderink, 1985, O’Regan and Noë, 2001). Nevertheless, there might or should be transsaccadic expectations for other object feature changes. The nature of such an expectation might strongly depend on or be determined by the compensation mechanism that the visual system uses to work around the processing limitations of peripheral vision. In other words, the build-up of transsaccadic expectations may be based on appearance of stimuli rather than on the earlier visual information. Recent findings by Cicchini et al. (2021) support this assumption demonstrating that visual priors in serial dependence are based on illusory stimulus properties rather than on physical ones. The authors also showed that those priors interact, however, with the physical rather than the illusory properties of a current stimulus. This complex interplay of prior expectations and stimulus appearance versus the early sensory information induced by it make stimulus features interesting that reveal an oppositional relationship between early versus later stimulus information and in foveal versus peripheral vision.

For example, high spatial frequency gratings are harder to make out in the periphery (e.g., Rovamo et al., 1992), reflecting a reduced availability of early, high spatial frequency information. On the other hand, spatial frequency has been shown to appear higher in the periphery compared to the fovea (Davis et al., 1987). Models on explaining the appearance difference across the visual field have been favouring a spatial-frequency channel-labelling mechanism (Davis et al., 1987, Davis, 1990). While these relationships would have to be confirmed by measuring pre- and postsaccadic appearance, a bias to perceive spatial frequency as higher in the periphery should lead to a transsaccadic expectation that predicts decreasing spatial frequency across saccades. It follows that changes that increase spatial frequency across saccades should be perceived more often. If this were true, it would also mean that transsaccadic expectations are built on appearance information rather than on low-level information. This hypothesis may be contradicted by Weiß et al. (2015), who did not report a bias in change detection for spatial frequency. However, since it was not the experimental goal of Weiß et al. (2015) to investigate a change-direction bias, the measurement applied in this study might not have been suited optimally for this purpose and further investigation may be needed here.

It may also well be, that the more complex a stimulus becomes i.e., the more feature dimensions the visual system can work with (e.g., colour + shape + luminance, or even feature combinations across modalities, see Stuckenberg et al., 2021), the more learned contingencies can be applied and compared to the incoming transsaccadic information. An accumulation of agreements with transsaccadic expectations for every feature may outweigh contradictions with transsaccadic expectations on spatial position such as large displacements or even blanks. This may become apparent in intrasaccadic displacement studies that found higher detection thresholds for naturalistic stimuli (McConkie & Currie, 1996); or a smaller blanking effect with complex stimuli (Tas et al., 2012, Stewart et al., 2020).

Finally, the influence of transsaccadic expectations may be manifold and become apparent not solely in conscious categorisation but also in reaction time (Huber-Huber et al., 2019, Stewart et al., 2020, Huber-Huber and Melcher, 2021) or, potentially, fixation duration (e.g. Henderson & Hollingworth, 2003), and pupil dilation (Preuschoff et al., 2011). Transsaccadic learning, that is the short-term learning of highly repetitive transsaccadic contingencies (e.g. Cox et al., 2005, Herwig and Schneider, 2014, Weiß et al., 2014, Valsecchi and Gegenfurtner, 2016), may also be affected by (long-term) transsaccadic expectations: on the one hand, larger prediction errors in one change direction might result in an increased updating of transsaccadic predictions and hence cause a larger learning effect (Rescorla & Wagner, 1972). One the other hand, larger prediction errors might be interpreted as evidence of object discrepancy as in causal-inference models (Körding et al., 2007, Atsma et al., 2016) and lead to a relatively weaker learning effect (Köller et al., 2020). Interestingly, in a transsaccadic-learning study that used the same shape stimuli as here, the participant group that experienced circularity increases across saccades showed an overall larger learning effect than the group that learned circularity decreases (Paeye et al., 2018, Experiment 2). However, it is unclear whether this difference between groups reflects a genuine difference in learning or whether it is due to differences in the baseline conditions between groups (judgements for unchanged objects). Furthermore, this difference was not always present (Paeye et al., 2018, Experiment 1). Further investigation would be needed to isolate an effect of long-term transsaccadic expectations on short-term learning of transsaccadic contingencies.

In summary, the character of transsaccadic expectations is likely to be specific for every visual feature dimension. Contradictions of and agreements with expectations in one feature dimension might affect change perception in general (for any other feature dimension) and may also be accumulated for or against external stability. In addition to change perception, transsaccadic expectations might affect several behavioural and perceptual measurements.

## 5 Conclusion

We found an overall shape-change direction bias for predominantly perceiving intrasaccadic shape changes that increased circularity across saccades. We further found that shape was perceived as more circular in presaccadic peripheral vision compared to postsaccadic foveal vision; but this appearance difference cannot directly explain the circularity-increase bias. We did, however, find a modulation of the overall bias on an inter-individual level presumably following from a direct but subsidiary influence of the appearance difference on the perceived magnitude of intrasaccadic shape changes. We conclude that the overall bias was due to an indirect influence of appearance differences across the visual field via a life-time learning of transsaccadic contingencies i.e., the built-up of transsaccadic expectations. This concept links transsaccadic perception of change or visual stability to a predictive-coding framework and implications following from this concept for other visual features in transsaccadic perception remain to be tested in the future.

## CRediT authorship contribution statement

**Carolin Hübner:** Conceptualization, Methodology, Software, Investigation, Formal analysis, Data curation, Writing - original draft, Visualization. **Alexander C. Schütz:** Conceptualization, Methodology, Supervision, Resources, Writing - review & editing, Funding acquisition.

## Declaration of Competing Interest

The authors declare that they have no known competing financial interests or personal relationships that could have appeared to influence the work reported in this paper.

## Footnotes

1 Note that this result would not be expected given the typical superiority of central compared to peripheral vison; but it was intended by our design as we halved the postsaccadic presentation duration in relation to the presaccadic presentation duration.

2 We use the term *expectation* to refer to implicit knowledge about the typical transsaccadic percept (the change from pre- to postsaccadic appearance resulting from a shift of feature information across the visual field). This knowledge should be acquired form life-time experience of such transsaccadic contingencies. The term *prediction* will be used to refer to the visual signal that is generated on demand by higher-level areas and that is based on presaccadic information and on transsaccadic expectations.

3 We use the term stability assumption not as a reference for a general tendency to assume stability but as one of two possible outcomes of the evaluation of transsaccadic information. The stability assumption may be a generalised phrase to refer to learned contingencies observed in our typically stable world, i.e., it represents the knowledge of re-occurring patterns of (visual) information resulting from eye- or body motion, and observations that are in accordance with this experience do not reach consciousness (the world remains stable).
