# Supplementary material for: A bias in saccadic suppression of shape change
Source: Vision Res. 2021 Sep;186:112–23. doi: 10.1016/j.visres.2021.05.005 (PMC7611036; doi:10.1016/j.visres.2021.05.005)
Supplement: Supplementary data 1 [file mmc1.docx]

## Supplementary material

Averaged median saccade latency over all participants was 151 ± 45 ms (mean ± standard deviation) for the no-blank condition and 152 ± 44 ms for the blank condition in Experiment 1A, and 159 ± 43 ms for the presaccadic condition and 199 ± 75 ms for the postsaccadic condition of Experiment 1B. Average saccade latency in Experiment 1A was also calculated for each change direction revealing an average saccade latency of 152 ± 46 ms for circularity-decrease and of 151 ± 42 ms for circularity-increase. According to Zimmermann et al. (2013) performance in intrasaccadic change detection increases with longer presaccadic observation time; hence, we wanted to control for this potential factor. We found no significant difference for saccade latencies between change direction (t(16) = 1.84, p = 0.085). In Experiment 2, median saccade latency averaged over all participants was 175 ± 53 ms for the circularity-decrease condition and 176 ± 54 ms for the circularity-increase condition in part A (non-significant difference, t(12) = -0.69, p = 0.5), and 180 ± 52 ms for the presaccadic condition and 188 ± 70 ms for the postsaccadic condition of part B.


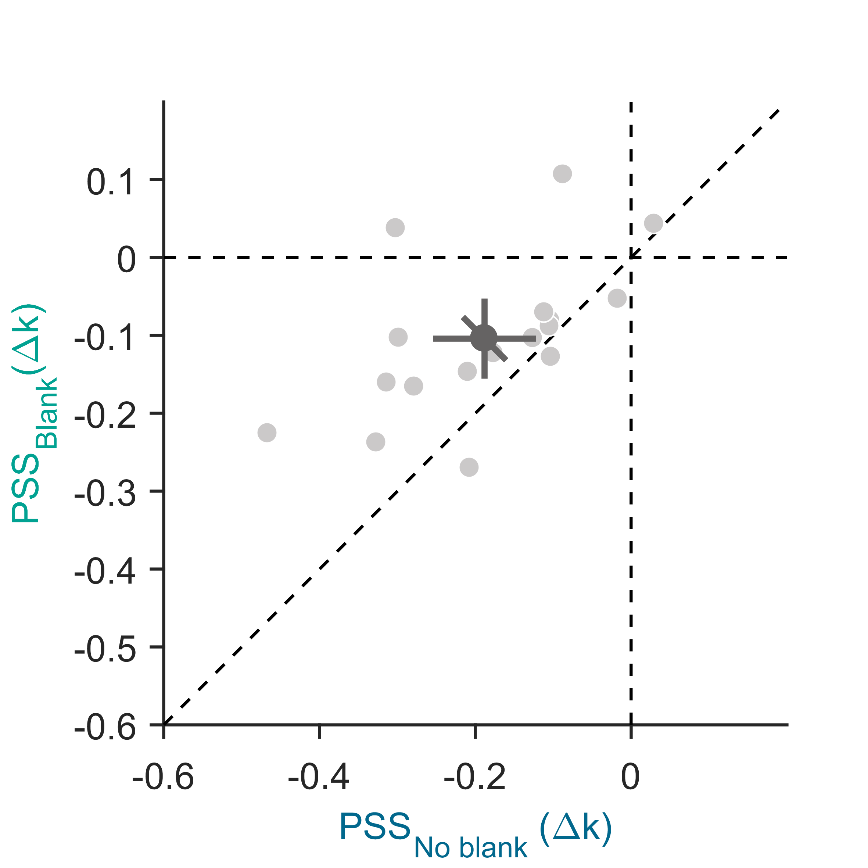


**Figure S1. Point of subjective stability (PSS) for perceived change magnitudes.** For this analysis, the value for the physical change magnitude of each trial was adjusted to correspond to a participant’s perceived change magnitude given their bias in pre- and postsaccadic conditions of Experiment 1B (difference between true mean and a participant’s PSE). This made changes of circularity-decrease numerically larger and changes of circularity-increase numerically smaller. It follows that the PSS bias for circularity-increase is even more pronounced (more negative) and it is now also significant for the blank condition. Data points on the dashed diagonal line indicate no difference between conditions. Light-grey dots represent individual participant data and the dark-grey dot indicates the overall mean. The error bars indicate 95%-confidence intervals within each condition (cardinal bars) or between conditions (oblique bar).

Additionally to the fit of full psychometric functions for Experiment 1A, the implemented sampling of shape changes (staircase procedure) enabled us to fit thresholds for both shape-change directions. Since participants directly reported shape-change direction, an analysis of thresholds in this paradigm would be inadmissible as an influence of a response bias cannot be excluded. Considering we could replicate the bias for circularity-increase changes with our criterion-fee paradigm in Experiment 2A, the influence of a response bias in the data of Experiment 1A seems unlikely; therefore, and while we make reservations regarding this analysis, we report individual thresholds for each change direction for the no-blank condition of Experiment 1A over pre- and postsaccadic appearance differences together with equivalent results for Experiment 2 (Figure S2). The results for Experiment 1 indicate, that the reduced shape-change bias observed for increased pre- and postsaccadic appearance differences (Figure 3E) may be due to a decrease in detection thresholds for circularity-decrease changes (slope m = -1.87, $p_{m}$ = 0.009, y-intercept n = 0.54, $p_{n}$ < 0.001, r² = 0.37) rather than to an increase in thresholds for the circularity-increase direction (m = 0.36, $p_{m}$ = 0.282, n = 0.18, $p_{n}$ < 0.001, r² = 0.08). Via an analysis of covariance we could confirm that the slopes of both linear fits were significantly different (F(1,30) = 10.81, p = 0.003). The observed relationship may underline that changes increasing circularity across saccades are of a different nature than changes in the opposite direction. Potentially due to the strong influence of transsaccadic expectations, detection thresholds for circularity-increase changes are relatively unaffected by appearance differences across the visual field and thresholds stay overall low. As can be seen in Figure S2, fits to the data of Experiment 2 show a similar tendency (shorter lines), however, the slope difference between fits was not significant (F(1,22) = 0.77, p = 0.389). This may be due to smaller variation across participants in pre- and postsaccadic appearance differences, which may be caused by the smaller sample size of Experiment 2.


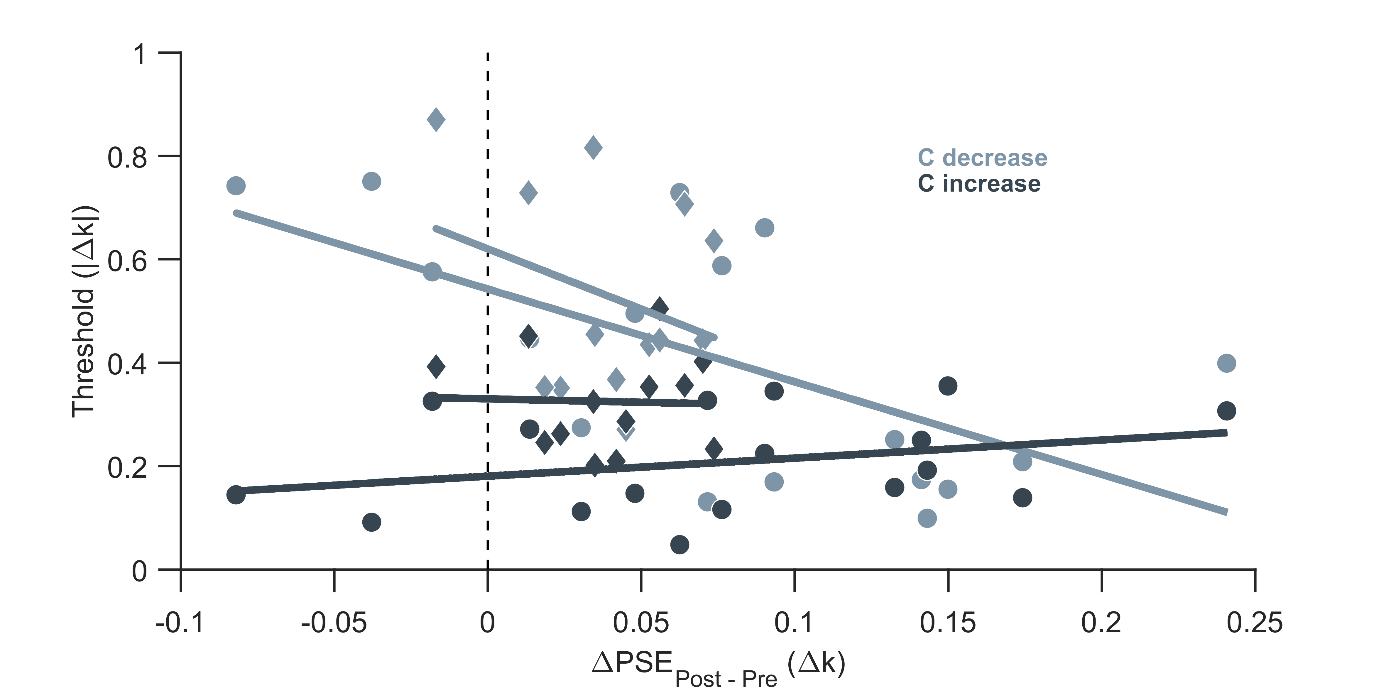


**Figure S2. Correlation analysis for change-detection thresholds from part A over appearance differences from part B for Experiments 1 and 2.** Thresholds (75%-correct) over PSE differences (postsaccadic minus presaccadic PSE) for circularity-decrease changes (light grey) and circularity-increase changes (dark grey) for Experiments 1 (circles represent individual data and long solid lines linear fits) and Experiment 2 (diamonds represent individual data and short solid lines linear fits).
